# Impact of real mirror profiles inside a split-and-delay unit on the spatial intensity profile in pump/probe experiments at the European XFEL

**DOI:** 10.1107/S1600577520014563

**Published:** 2021-01-01

**Authors:** V. Kärcher, S. Roling, L. Samoylova, A. Buzmakov, U. Zastrau, K. Appel, M. Yurkov, E. Schneidmiller, F. Siewert, H. Zacharias

**Affiliations:** a Physikalisches Institut, Westfälische Wilhelms-Universität, 48149 Münster, Germany; b European XFEL GmbH, 22869 Schenefeld, Germany; c FSRC ‘Crystallography and Photonics’ RAS, 119333 Moscow, Russia; d Deutsches Elektronen-Synchrotron, 22603 Hamburg, Germany; e Helmholtz-Zentrum Berlin für Materialien und Energie, Department Optics and Beamlines, 12489 Berlin, Germany; f Center for Soft Nanoscience, Westfälische Wilhelms-Universität, 48149 Münster, Germany

**Keywords:** free-electron laser, pump/probe experiment, split-and-delay unit, hard X-rays, compound refractive lenses

## Abstract

For the High-Energy-Density (HED) beamline at the SASE2 undulator of the European XFEL, a hard X-ray split-and-delay unit (SDU) has been built enabling time-resolved pump/probe experiments with photon energies between 5 keV and 24 keV. The optical layout of the SDU is based on geometrical wavefront splitting and multilayer Bragg mirrors. Maximum delays between Δτ = ±1 ps at 24 keV and Δτ = ±23 ps at 5 keV will be possible. Time-dependent wavefront propagation simulations were performed by means of the *Synchrotron Radiation Workshop* (*SRW*) software in order to investigate the impact of the optical layout, including diffraction on the beam splitter and recombiner edges and the three-dimensional topography of all eight mirrors, on the spatio-temporal properties of the XFEL pulses. The radiation is generated from noise by the code *FAST* which simulates the self-amplified spontaneous emission (SASE) process. A fast Fourier transformation evaluation of the disturbed interference pattern yields for ideal mirror surfaces a coherence time of τ_c_ = 0.23 fs and deduces one of τ_c_ = 0.21 fs for the real mirrors, thus with an error of Δτ = 0.02 fs which is smaller than the deviation resulting from shot-to-shot fluctuations of SASE2 pulses. The wavefronts are focused by means of compound refractive lenses in order to achieve fluences of a few hundred mJ mm^−2^ within a spot width of 20 µm (FWHM) diameter. Coherence effects and optics imperfections increase the peak intensity between 200 and 400% for pulse delays within the coherence time. Additionally, the influence of two off-set mirrors in the HED beamline are discussed. Further, we show the fluence distribution for Δ*z* = ±3 mm around the focal spot along the optical axis. The simulations show that the topographies of the mirrors of the SDU are good enough to support X-ray pump/X-ray probe experiments.

## Abstract 2

This paper describes a detailed simulation of the influence of optical imperfections in a multilayer Bragg mirror-based split-and-delay unit on the focused and unfocused beam profiles with relative time delays within the coherence time for hard X-ray pump/X-ray probe experiments at the European XFEL.

## 1. Introduction   

In the past decade, the investigation of ultrafast processes on the spatial nanoscale and at ångström wavelengths has gained increasing interest, for which a suitable light source with well defined temporal and spatial properties is required. Free-electron lasers (FELs) provide widely tunable and femto­second X-ray light pulses, paving the way for the investigation of the dynamics of nanoscale objects on the pico- and femtosecond timescale. Generated by self-amplified spontaneous emission (SASE), the pulses are characterized by a well defined wavefront with a high degree of coherence compared with common synchrotron radiation sources (Schlotter *et al.*, 2010 ▸; Roling *et al.*, 2011 ▸; Singer *et al.*, 2012 ▸). Presently, there are five operating FELs generating partially coherent X-ray radiation (Altarelli *et al.*, 2006 ▸; Kim *et al.*, 2008 ▸; Emma *et al.*, 2010 ▸; Abela *et al.*, 2017 ▸; Tanaka & Yabashi, 2012 ▸). The European XFEL accelerates electron bunches to energies of up to 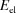 = 17.5 GeV and generates photon energies between 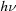 = 3 keV and 24 keV in the SASE1 and SASE2 undulators. Up to 27000 pulses with pulse durations between 2 fs and a few hundred femtoseconds and pulse energies at saturation of up to 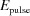 = 2 mJ at 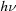 = 5 keV are generated at a time interval of 220 ns (∼4.5 MHz) and a burst repetition rate of 10 Hz (Altarelli *et al.*, 2006 ▸). A femtosecond time resolution required for the investigation of ultrafast dynamic processes is not provided by the European XFEL itself. Such a time resolution can be achieved by optical pump/X-ray probe set-ups when the dynamics is induced by valence band electrons. Dynamic processes initiated by inner-shell electrons can be studied at FELs with an accelerator-based two-bunch mode (Hara *et al.*, 2013 ▸; Lutman *et al.*, 2013 ▸; Marinelli *et al.*, 2015 ▸), or by means of a split-and-delay unit (SDU) (Roseker *et al.*, 2009 ▸; Sorgenfrei *et al.*, 2010 ▸; Sobierajski *et al.*, 2013 ▸; Wöstmann *et al.*, 2013 ▸; Hilbert *et al.*, 2014 ▸; Roling *et al.*, 2014*a* ▸) which may yield elemental specificity to both X-ray pump and the X-ray probe. In the latter case, the pulse will be separated into two partial beams which can be delayed with respect to each other by a variation of their path length. Such devices for hard X-ray radiation enable time-resolved coherent diffractive imaging (CDI) with a sub-nanometre resolution on the pico- and femtosecond timescale (Neutze *et al.*, 2000 ▸; Rath *et al.*, 2014 ▸). Furthermore, nonlinear effects within the coherence time in pump/probe ion spectroscopy experiments were demonstrated (Jiang *et al.*, 2010 ▸; Ding *et al.*, 2019 ▸). Hence, knowledge about the spatial and temporal beam properties is required for a precise determination of the beam intensity profile at the target position and for a correct interpretation of the results.

Split-and-delay units make use either of amplitude splitting or of geometrical wavefront splitting. Most current SDUs for hard X-ray radiation use single crystals in Bragg and Laue geometry (Roseker *et al.*, 2009 ▸; Zhu *et al.*, 2017 ▸; Hirano *et al.*, 2018 ▸; Lu *et al.*, 2018 ▸; Sun *et al.*, 2019 ▸; Rysov *et al.*, 2019 ▸). However, such crystals transmit only a narrow bandwidth of 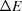 = 0.5 eV (Roseker *et al.*, 2009 ▸) which not only will enhance the temporal coherence properties due to the Wiener–Khintchine theorem but due to the special geometry of the crystal the intensity of the pulse is significantly reduced. A certain type of experiment requires such conditions (Grübel & Zontone, 2004 ▸; Chapman *et al.*, 2007 ▸; Roseker *et al.*, 2018 ▸, 2020 ▸), but nonlinear interactions severely suffer and a determination of coherence properties of the complete SASE spectrum is not possible with crystal-based devices. The latter cases require the transmittance of the complete spectrum such that the temporal beam properties are conserved. Currently, this is realized in two SDUs based on geometrical wavefront splitting for wavelengths in the XUV regime which are now operating at the BL2 and PG2 beamline at FLASH in Hamburg (Sorgenfrei *et al.*, 2010 ▸; Wöstmann *et al.*, 2013 ▸). With these devices, jitter-free time-resolved pump/probe experiments have been realized (Krikunova *et al.*, 2012 ▸; Zastrau *et al.*, 2014 ▸).

The novel SDU based on multilayer Bragg mirrors (Kazimirov *et al.*, 2006 ▸; Roling *et al.*, 2014*a* ▸) will be integrated into the HED beamline of the SASE2 undulator at the European XFEL. It enables time-resolved two-pulse correlated hard X-ray experiments at high intensities (Roling *et al.*, 2012 ▸). One of the challenges in this regime is the conservation of the beam properties while propagating over multiple reflective and refractive optics. Real mirrors do not show ideal, flat mirror surfaces. Thus, the hard X-ray pulses are reflected by surfaces which show height variations significantly larger than the wavelength resulting in a distortion of the beam profile. Such impacts on the beam profile can be investigated by numerical simulations of wavefront propagation. Generally, these beam properties are influenced by optics imperfections and in cases of a wavefront splitting SDU by the diffraction at the mirror edges and by the relative time delay between the two separated partial beams. For delays within the coherence time, the overlap of the two pulses leads to the formation of interference fringes, which is characterized by a periodically modulated intensity. This intensity distribution will be altered by real mirror shapes used in the SDU thus forcing the beam profiles to be disturbed. A quantitative and predictive understanding of these effects, essential for the evaluation of pump/probe experiments with delays below the coherence time, requires the following three prerequisites. First, the properties of the optical elements within the beam path have to be known, such as the mirror surface profiles (Siewert *et al.*, 2011 ▸, 2012 ▸, 2013 ▸). Second, the knowledge of the spatial and especially the temporal coherence properties, *e.g.* from a measurement by means of an SDU, is required. Third, an in-depth evaluation of the influence of both on the focused beam profile by means of wavefront propagation simulations is necessary.

Here we present results of the propagation of a SASE pulse at a photon energy of 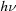 = 4.96 keV through this SDU. In the first simulated set-up the split-and-delay unit with all eight mirrors is placed in the propagation path without any focusing elements. Previously we carried out such simulations using only 1D height profiles of only two mirrors (Roling *et al.*, 2014*b* ▸). Now the simulation will include the complete 3D profiles of all eight mirrors of the SDU. Further the two grazing-incidence beam offset mirrors of the SASE2 beamline will be taken into account. This yields a meaningful estimation of the transverse fluence distribution – the beam profile – at the target position. In the second simulated configuration compound refractive lenses (CRLs) are included as focusing elements in the propagation path. The fluence and phase distribution at the focal spot will be used as input data for start-to-end simulations of single particle imaging and scattering experiments (Fortmann-Grote *et al.*, 2017 ▸). Furthermore, the longitudinal fluence distribution around the focal spot will be discussed to obtain an estimation of the imperfections in propagation direction. This SDU provides additionally tunable two-color multilayer Bragg mirrors (Roling *et al.*, 2014*a* ▸) and will be installed in the year 2021 at the European XFEL. Hence, these results will be important to all experimentalist to design experiments.

## 2. The split-and-delay unit for the HED instrument   

**Figure 1:**
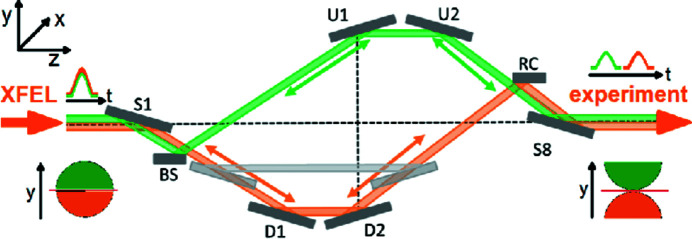
Schematic drawing of the optical layout of the hard X-ray split-and-delay unit (Roling *et al.*, 2012 ▸).

The optical layout of the SDU is depicted in Fig. 1 ▸. All mirrors are coated by multilayer stacks of *M*B_4_C (Roling *et al.*, 2014*a* ▸), where *M* denotes Ni, Mo or W, which allows steeper grazing angles and thus longer temporal delays between both pulses. That requires, however, reflection at the Bragg condition, which yields comparatively large grazing angles at low photon energies (5 keV) and significantly shallower ones at high photon energies (24 keV). The incoming beam is then reflected by the first mirror S1 at the Bragg condition. One part of the reflected beam (green) hits the beam splitter BS while the other part (orange) passes the edge of the BS. Thus, the splitted partial beams are propagating through different paths. For both branches, the path length can be adjusted by moving the mirror pairs D1 and D2 or U1 and U2 along the propagation path in order to tune the required time delay between the partial beams. At the exit of the SDU the situation is reversed due to the point symmetrical layout. Now, the partial beam depicted in orange is reflected by the recombiner RC and the green part passes the edge of RC. The last mirror S8 reflects both partial beams into the initial propagation direction. The wavefronts of both partial beams are rotated by 180° due to the odd number of reflections.

For photon energies of 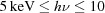 keV the multilayer Bragg mirrors BS and RC are coated by 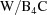 stacks. Using the Bragg condition 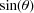 = 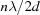, where *n* is the order of diffraction, the grazing angle θ for a pulse of 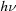 = 5 keV is 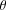 = 3.6° resulting from a stack thickness of 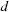 = 1.948 nm. All other mirrors used in this photon energy range are made of 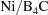 stacks with a period of 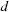 = 4 nm, resulting in a grazing angle of 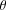 = 1.8° at 5 keV. For photon energies above 10 keV 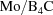 multilayers with a period of 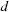 = 1.6 nm and 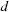 = 3.2 nm are used for BS/RC and the other mirrors, respectively (Roling *et al.*, 2014*a* ▸).

**Figure 2:**
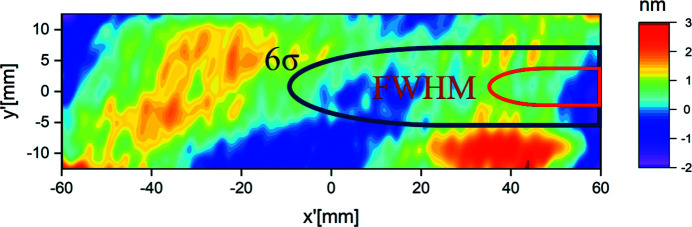
Topography of the beam splitter. The 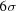 (blue) and the FWHM (red) footprint at 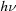 = 5 keV are indicated. Within the blue and red labeled area the maximum height deviations amounts to 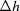 = 3 nm.

The multilayer Bragg mirrors do not show ideal, flat surfaces. Thus, the hard X-ray pulses are reflected by surfaces which show height variations on the order of the wavelength. The resulting wavefront distortions 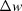 can be described as (Roling *et al.*, 2014*b* ▸; Samoylova *et al.*, 2016 ▸) 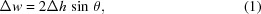
or after inserting the Bragg conditions for 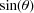 in units of λ,
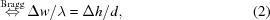
where 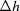 is the local surface height error. This indicates that the relative distortions are independent of the photon energy. It is a direct measure of the properties and quality of the mirrors. For the beam splitter (BS) the measured height topography is shown in Fig. 2 ▸ with a maximal peak-to-valley error of 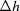 = 5 nm over the whole surface. The surface profiles of all mirrors are shown in Figs. A1.1 to A1.7 in the supporting information. The maximal peak-to-valley values are in the range 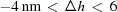 nm. At a photon energy of 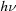 = 4.96 keV, which corresponds to a wavelength of 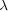 = 0.25 nm, the grazing angle of incidence at the BS amounts to 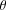 = 3.6°. Then, the maximal beam distortion over the whole mirror surface would be 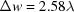. At this photon energy the direct FEL beam diameter is expected to be 3.27 mm (FWHM) and 8.33 mm (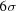) at the position of the SDU. Then, the 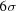 footprint of the full beam extends to 132 mm. Since the beam will be cut at the edge of the BS, the irradiated part of the beam splitter ranges from −6 mm to 60 mm for a splitting ratio of 1:1, as indicated in Fig. 2 ▸. Within this area, the maximal peak-to-valley error is reduced to 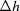 = 3 nm causing a distortion of about 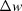 = 1.5λ. For any complex field 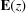 this wavefront distortion 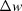 will cause scattered light waves resulting in the appearance of speckles at the sample position. These speckles appear at spatial frequencies 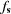 which can be estimated to (Samoylova *et al.*, 2016 ▸) 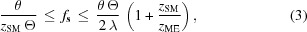
where 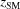 is the source-to-mirror distance, 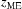 is the mirror-to-experiment distance and Θ is the divergence of the beam. In the case of the BS for which 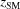 = 846 m and 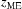 = 126 m, the spatial frequencies of the distorted wavefront range between 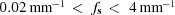 for 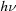 = 4.96 keV and 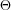 = 3.87 µrad (FWHM).

Since the experiments which will be performed at the HED instrument require intensities on the order of 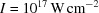, the X-ray pulses will be focused. Thus, we further investigate the ability to focus the distorted beams including CRLs in the propagation path. Naturally, all optical elements introduce some distortions. A measured example for the distortion of a wavefront caused by one parabolic-shaped CRL for a wavelength of 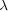 = 0.054 nm (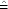 23 keV) is 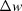 = 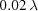 (Rutishauser *et al.*, 2011 ▸). According to equation (1), the wavefront distortion 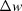 caused by the beam splitter for 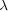 = 0.054 nm (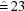 keV) amounts for an incidence angle of 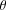 = 0.2° and 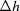 = 3 nm to 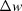 = 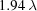. Thus, the impact of the mirrors of the SDU will be most significant. Since the footprint reduces with increasing photon energies, the effective wavefront distortion will be largest at low photon energies.

## 3. Methods   

In order to investigate the spatio-temporal properties of XFEL pulses by means of numerical simulations, two steps have to be taken into account. First, the radiation has to be generated under the conditions of the SASE process. Second, the radiation has to be propagated through X-ray specific components like grazing-incidence plane mirrors and CRLs. The generation of the radiation from shot noise is realized by means of the code *FAST* (Saldin *et al.*, 1999 ▸). A SASE pulse that is generated in this way can be used as an input for the *SRW* package (Chubar *et al.*, 2008 ▸; Samoylova *et al.*, 2011 ▸, 2016 ▸). The *SRW* package is a Fourier optics based approach which has proven its utility in a large number of applications (Chubar *et al.*, 2008 ▸). It includes all optical components as numerical propagators. The *WPG* tool (Samoylova *et al.*, 2016 ▸) provides high-level access to the *SRW* functions by means of a Python script.

### 3.1. Numerical methods   

At the very beginning of the undulator, electrons with different phases enter the undulator at different times. Thus, the radiation is generated at different times *t* and the electric fields are calculated in the time domain. The associated frequencies ω are directly connected to the time domain by means of the Fourier transformation 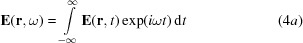
and 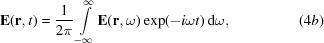
where 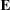 denotes the electric field. Then, the generated radiation starts to interact with the electrons inducing a correlated ordering in the electron bunches. Hence, the generated radiation will be correlated, too. The coherence properties increase until the radiation power is saturated. In the saturated regime of a SASE FEL the longitudinal and transverse coherence properties reach their highest level, and they decrease directly afterwards (Saldin *et al.*, 2008 ▸). Thus, a saturated SASE spectrum is used for the simulations. The spatial mode is a 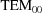 mode and propagates as a divergent Gaussian beam. With this as an input, the generated field 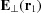 at a position 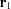 can be propagated in vacuum via the Huygens–Fresnel principle in order to calculate the field 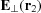 at a position 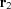 in the frequency domain, 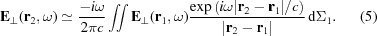
Since the integration plane 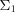 will be perpendicular to the propagation direction 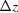, equation (5) becomes a convolution integral with 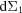 = 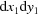 and 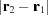 = 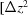 + 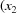 − 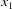)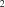 + 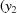 − 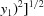 which can be solved by means of a 2D fast Fourier transformation (FFT) algorithm. A detailed description of the propagators has been given by Samoylova *et al.* (2016 ▸).

### 3.2. Intensity distribution of undistorted beam profiles   

In pump/probe experiments with a temporal separation of both pulses larger than the pulse duration, only the beam profile of the individual beams matter. However, for shorter temporal separations the spatial and temporal coherence properties of the FEL radiation have to be taken into account for a correct description of the intensity profile and a correct interpretation of experimental results (Ding *et al.*, 2019 ▸). Saturated SASE FEL pulses are characterized by partially coherent radiation. In particular, the high degree of transverse coherence is an outstanding feature (Saldin *et al.*, 2008 ▸). Both transverse and longitudinal coherence can be measured with an SDU. To perform pump/probe experiments with time delays shorter than the coherence time or for a measurement of the temporal coherence both partial beams are overlapped non-collinearly at a target or on a detector under a fixed overlapping angle. At different delays τ interferences occur which can be recorded within the coherence time or pulse duration. For a measurement of the spatial coherence properties the delay is kept fixed at 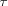 = 0 fs and the distance between the points 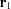 and 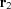 is changed. The theoretical description of an interaction between two fields is given by a time-averaged multiplication of the two fields which is defined as the mutual correlation function Γ (Goodman, 1985 ▸; Mandel & Wolf, 1995 ▸),
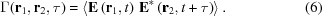
Equation (6) can be written in a normalized form. For a fixed distance 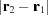 = const, the correlation function becomes 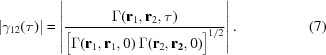
Equation (7) can be calculated by means of the visibility *V* which is defined as 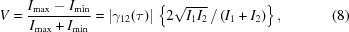
where 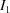 and 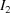 denote intensities of the interfering partial beams, and 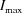 and 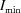 are the maximum and minimum intensities of the interference fringes. Using the definition of the visibility according to equation (8), the intensity at the target or on the detector, 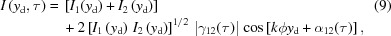
can be directly related to the complex degree of coherence, where 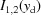 = 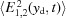, *k* is the wavenumber and 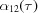 is the phase of the complex degree of coherence. The first term describes the intensities of the two splitted beams. In the second term, a cosinusoidal modulation is introduced. Its modulation depth is given by the correlation function 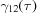. It is necessary to overlap the two partial beams for a fixed relative vertical distance at S8 by introducing the slightly different angle of incidence 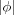 for the second beam on the vertical detector position 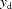. Thus, the interference fringes at the sample or on the detector which is positioned at a distance *z* behind the SDU will show a spatial period length of 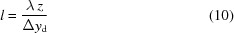
where 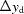 is the variable overlap region. For two fixed points 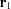 and 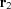, the temporal coherence 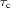 can be described by 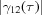 as a function of 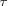. Definitions of 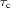 are the half width at half-maximum (HWHM) which is used in this evaluation or the width at a value of 1/e of the maximum of a Gaussian function fitted to 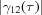.

The determination of the coherence time by calculating the visibility is strictly only possible if solely time-dependent two-beam interference causes the formation of fringes. Additional well defined fringes that may occur for instance from diffraction at the edge of the beam splitter can still be tolerated in the case of an overlap for fixed points 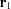 and 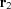. However, arbitrary distortions caused by the imperfections of the mirror surfaces make a determination of the coherence time by means of equation (8) not suitable. Hence, a different approach has to be applied. After the measurement of the intensity distribution according to equation (9), its Fourier transformation yields 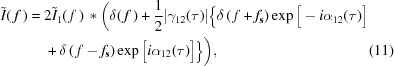
where *f* denotes the spatial frequencies in general, δ is the Dirac delta function, 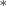 is the convolution operator and 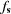 is the spatial frequency of the interference fringes. The signal originating from time-dependent two-beam interference then occurs at this well defined spatial frequency 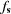 = 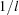, compare equations (9) and (10). The signal 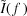 decreases for increasing temporal delays, while the signal caused by wavefront distortions is spread over a wide spatial frequency range and stays constant for large delays. By normalizing the signal of the Fourier transform 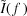 for different delays τ to the signal at zero delay, the coherence time 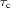 can be determined. This method of evaluation was first employed by Schlotter *et al.* (2010 ▸).

## 4. Time-dependent wavefront propagation simulation   

**Figure 3:**
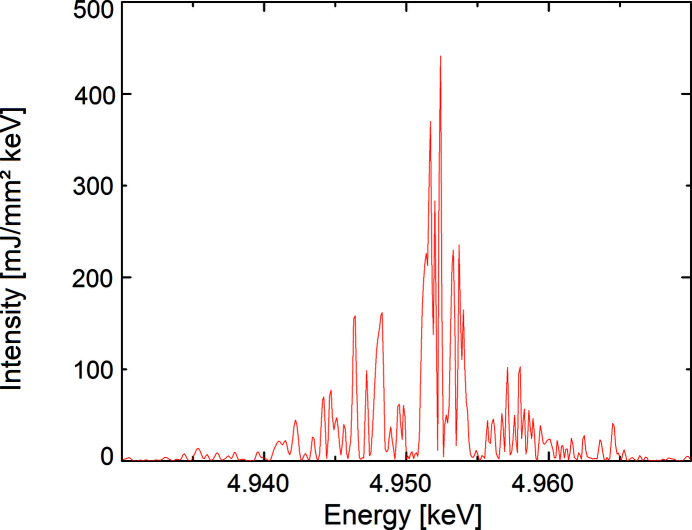
The spectrum of the FEL pulse that was used for the wavefront propagation simulation.

In this section the propagation of the wavefront through optical components of the SASE2 beamline is described for a photon energy of 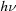 = 4.96 keV. Taking into account the operating condition of the SASE2 undulator (Nakatsumi *et al.*, 2014 ▸), electrons with a charge of 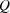 = 100 pC are accelerated to relativistic energies of 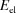 = 11.5 GeV by applying the code *FAST* (Saldin *et al.*, 1999 ▸). The power density is generated in the time domain. Since the propagation of the wavefront takes place in the frequency domain, the spectrum has to be calculated. The generated SASE spectrum with its typical spiky structure is shown in Fig. 3 ▸. The transverse profile of the radiation corresponds to a Gaussian 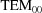 mode (Geloni *et al.*, 2010 ▸) leaving the undulator with a divergence of 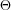 = 3.87 µrad (FWHM). Higher-order spatial modes may also occur in reality, which may disturb the interference pattern. But at the end of the linear regime in an undulator these modes are mostly suppressed (Geloni *et al.*, 2010 ▸).

Leaving the undulator, the linearly polarized beam propagates towards the optical components. Here, two different configurations are simulated. In the first configuration the beam propagates directly towards the SDU. Its first mirror (S1) is located at 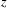 = 846 m downstream from the source, and the beam diameter amounts to 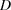 = 3.27 mm (FWHM) and 8.33 mm (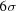). The BS splits the beam in a 1:1 ratio in the vertical direction generating two equally shaped partial beams with a diameter of 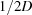 = 1.63 mm (FWHM). The partial beams hit the surface of the BS and RC at a grazing angle of 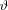 = 3.6° causing a footprint of 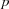 = 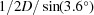 = 24 mm (FWHM) and 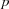 = 66 mm (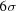) as indicated in Fig. 2 ▸. The incident angle for the other six mirrors amounts to 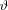 = 1.8°. Thus, the footprint doubles in length. The exit of the SDU is located at 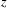 = 852 m. The sample position is at 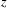 = 972 m. Thus, the partial beams propagate 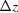 = 120 m until they reach the experiment. Note that in the beamline two additional off-set mirrors (OM1, OM2) are positioned at 290 m and 301 m (Nakatsumi *et al.*, 2014 ▸), but the impact of the height and slope errors of the SDU are more significant.

**Figure 4:**
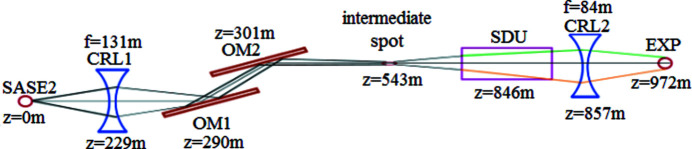
The complete simulated configuration that was derived from Nakatsumi *et al.* (2014 ▸).

In a second configuration, which is derived from the technical design report of the European XFEL (Nakatsumi *et al.*, 2014 ▸), the beam propagates towards a first CRL system at *z* = 229 m which prefocuses the beam with a focal length of 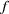 = 131 m generating an intermediate focal spot at 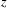 = 543 m. This reduces the beam diameter at the SDU to 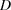 = 0.99 mm (FWHM). Consequently, only 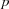 ≃ 8 mm (FWHM) and 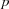 ≃ 22 mm (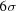) of the beam splitter (BS) and recombiner (RC) are irradiated by each partial beam, and twice as much for the other mirrors. A second CRL system is positioned at 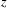 = 857 m with a focal length of 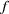 = 84 m. The focal spot is at the sample position. All optical components with their positions along the optical axis *z* are listed in Table 1 ▸. The second simulated configuration is depicted in Fig. 4 ▸ where we include the two off-set mirrors OM1 and OM2. We neglect the distribution mirror positioned at 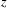 = 390 m.

**Figure 5:**
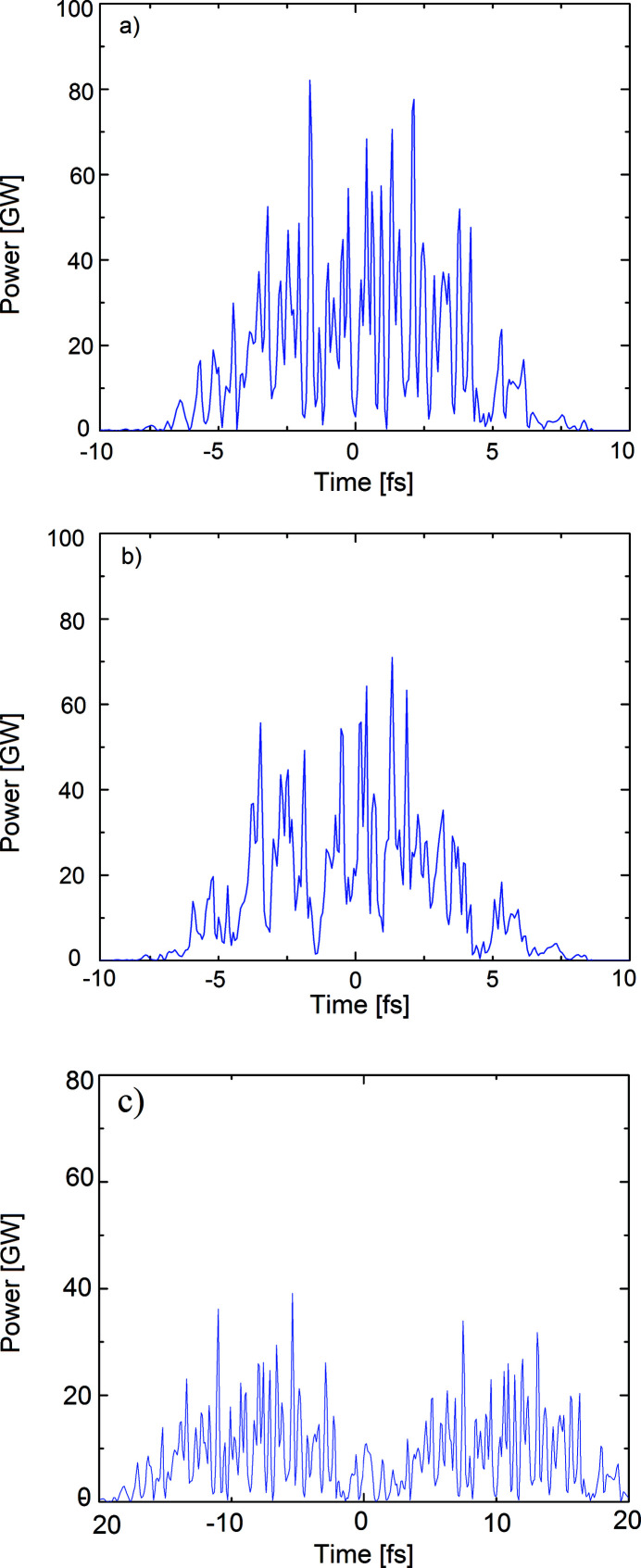
The temporal shape of the FEL pulse at 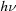 = 4.96 keV for (*a*) τ = 0 fs, (*b*) τ = 0.24 fs and (*c*) τ = 13 fs where the splitted pulses are clearly separated.

After the propagation through all optical components, the electric fields are recalculated in the time domain. Fig. 5 ▸ displays the power distribution with its stochastically spiky structure for different delays τ in the time domain. For 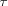 = 0, the power distribution is displayed in Fig. 5 ▸(*a*). The pulse duration amounts to 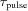 ≃ 10 fs. The width of one spike is of the order of the coherence time 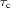 of the pulse. In this simulation the coherence time of the pulse is 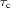 = 0.243 fs (r.m.s.). Figs. 5(*b*) and 5(*c*) ▸ show the power distribution for delays of 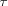 = 0.24 fs and 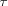 = 13 fs, respectively. In the latter case, the pulses are clearly separated.

### 4.1. Effects of mirror imperfections on focused pump/probe XFEL pulses   

**Figure 6:**
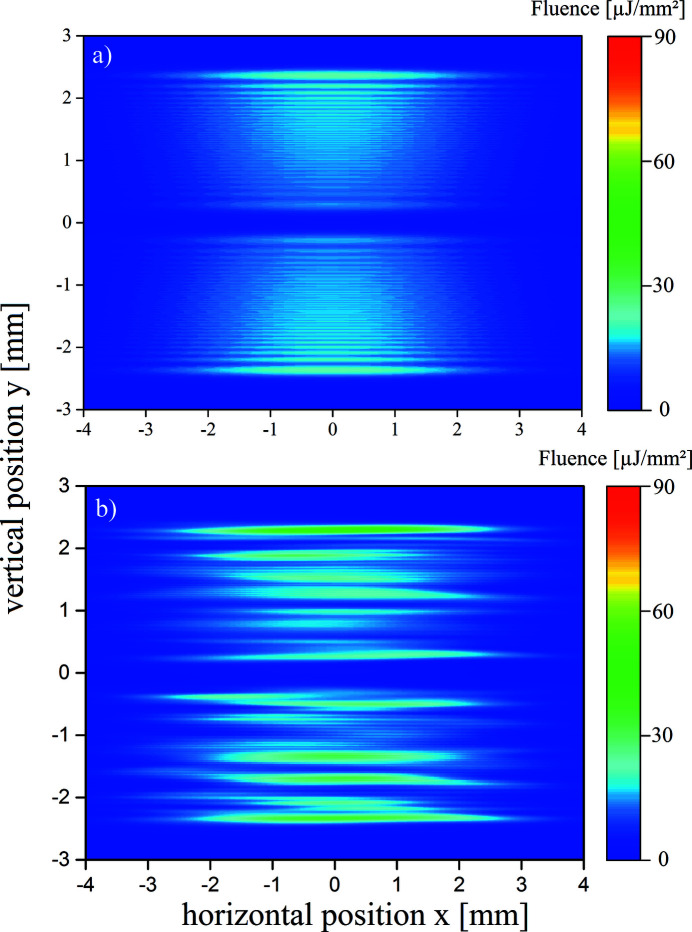
The separated transverse profile of both partial beams on an imaging detector at *z* = 972 m containing (*a*) ideally flat and (*b*) real mirrors. The FWHM in horizontal direction is 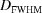 = 5.2 mm.

The SDU separates the beam by geometrical wavefront splitting. For the splitting ratio of 1:1 this causes two equally shaped fluence distributions with an additional spatial modulation due to the diffraction at the mirror edges of the BS and RC. Fig. 6 ▸ shows the transverse profile at the target position at 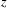 = 972 m for unfocused beams in case of (*a*) ideal mirror surfaces (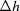 = 0) and in case of (*b*) real mirror surfaces (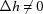). In both cases, the modulation that is caused by the edges of the BS and RC is recognized. However, the fluence distribution shown in Fig. 6 ▸(*b*) is obviously distorted due to the finite height variations of the mirrors. Furthermore, the partial beams are turned around by 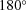 due to the point symmetric set-up of the SDU.

**Figure 7:**
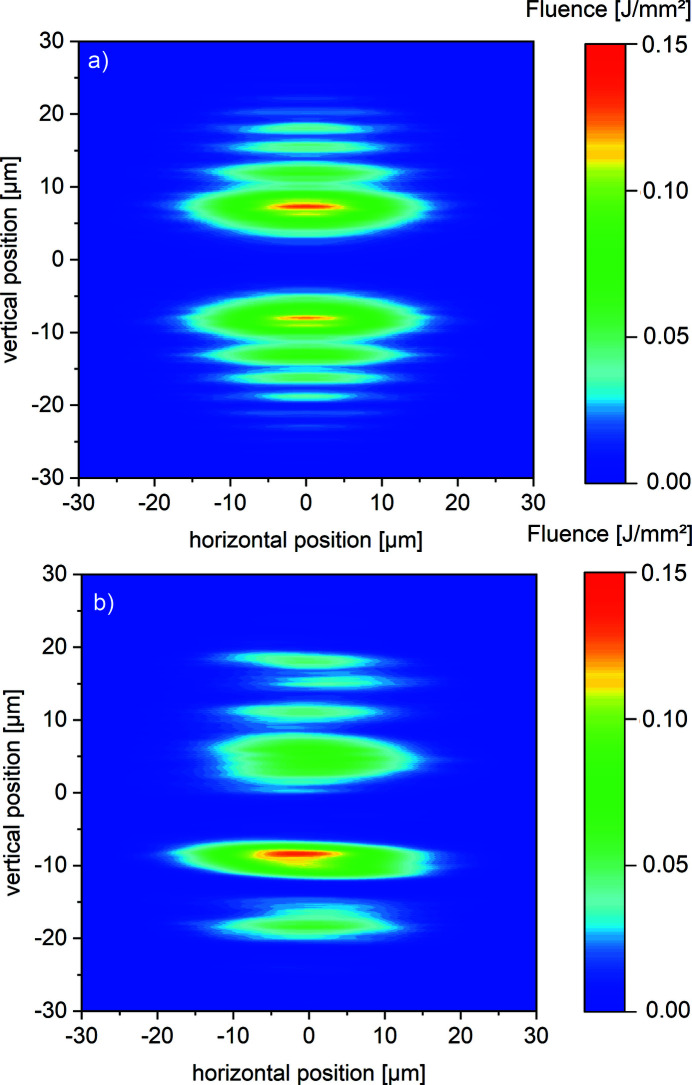
The separated transverse profiles on an imaging detector located at the focal spot at *z* = 972 m containing (*a*) ideal flat and (*b*) real mirrors for one possible lens configuration in the HED tunnel.

In order to investigate the impact of the wavefront distortions caused by the mirror imperfections on the focal spot, a second configuration including the SDU and CRLs is simulated. This lens configuration provides a Rayleigh length of 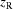 = 5 m. The first lens (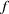 = 131 m) which is located at 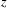 = 229 m downstream from the source prefocuses the beam, see Fig 4 ▸. This reduces the footprint on the mirrors inside the SDU by a factor of three. The second lens (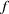 = 84 m) is located 5 m behind the SDU at 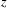 = 857 m and generates a spot width of 20 µm (FWHM) at the target. Fig. 7 ▸ shows the transverse profiles for spatially separated partial beams. The case of ideal mirror surfaces inside the SDU is depicted in Fig. 7 ▸(*a*). A main lobe with a vertical width of 6 µm for both partial beams and a clear modulation due to diffraction at the edges of the beam splitter and recombiner is obtained. Note that the beams are turned around by 180° once again. The intense parts of both partial beams with fluence values of I = 0.11 J mm^−2^ are close together now. The fluences of the maxima decrease smoothly and evenly along the vertical and horizontal axis as is shown in Fig. 7 ▸. Furthermore, there are no distortions caused by the lenses themselves, as expected for homogeneous media. Thus, the effect of the real mirror height profiles on the ability to focus the beam can be studied. The effect of these height variations for the same focal region as in the ideal case is depicted in Fig. 7 ▸(*b*). The modulation occurring due to diffraction at the edges of the mirrors BS and RC is still present but disturbed. A comparison of both partial beams reveals that the fluence is no longer evenly distributed as in Fig. 7 ▸(*a*) and that the spatial shape of one pulse differs from that of the other. The lower partial beam in Fig. 7 ▸(*b*) was propagated via the mirrors S1, D1, D2, RC and S8, and the upper via S1, BS, U1, U2 and S8. The lower partial lobe shows an intense part with fluence values up to 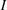 = 0.15 J mm^−2^ and a vertical width of 4 µm and a less intense second lobe with 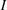 = 0.07 J mm^−2^ and a vertical width of 6 µm. In the upper partial beam the fluence is relatively equal distributed with peak values about 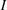 = 0.07 J mm^−2^ with a width of 6 µm for the largest lobe. The simulation demonstrates clearly that the lower partial beam propagated via D1 and D2 should be used as a pump pulse in pump/probe experiments.

**Figure 8:**
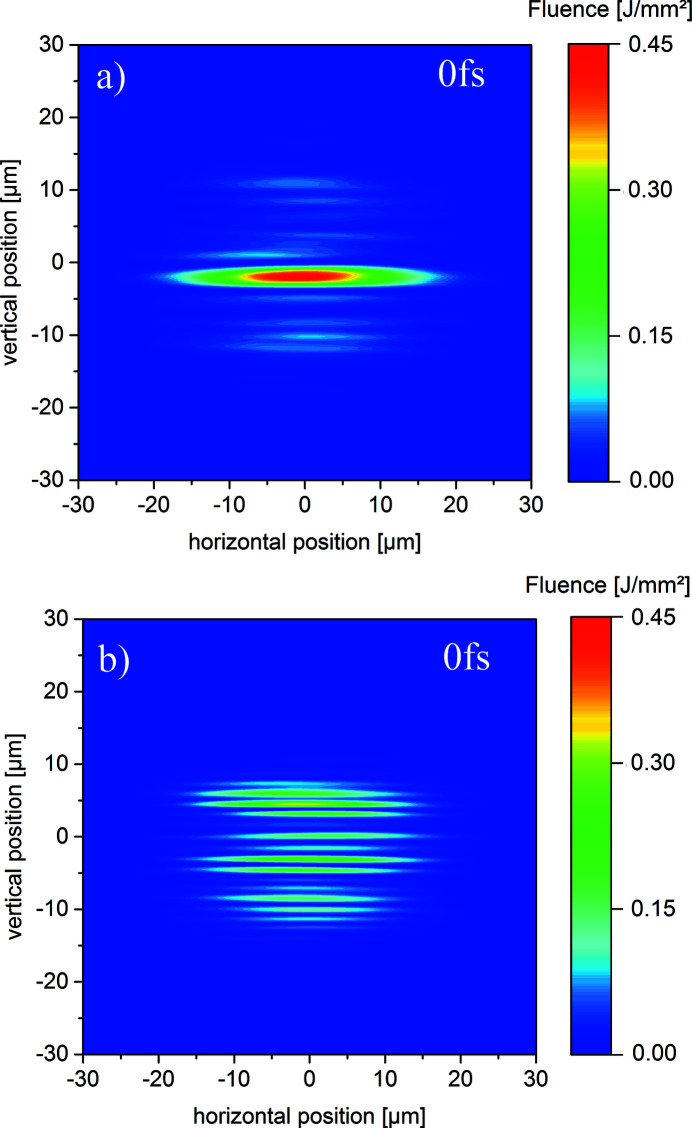
The transverse profiles on an imaging detector located at the focal spot at *z* = 972 m overlapping the intense parts in (*a*). The focal spot for a complete overlap is depicted in (*b*) using one possible lens configuration in the HED tunnel. In both cases, real mirror profiles are used.

Two overlap regions for 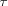 = 0 fs are depicted in Fig. 8 ▸ for the case of real mirrors only. In Fig. 8 ▸(*a*) only the intense parts of both beams with an FWHM value of 3 µm are overlapped with a maximal fluence of *I* = 0.45 J mm^−2^ which is three times larger compared with the separated case depicted Fig. 7 ▸(*b*). This increase is caused by constructive interference within the coherence time of both pulses. Using ideal flat mirrors the fluence of this peak is about 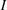 = 0.38 J mm^−2^. This picture is provided in Fig. A2.1 of the supporting information. Hence, not only the ability to form interference fringes for time delays of 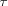 < 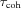 but also the 3D topography of the mirrors of the SDU can increase the intensity which may be important for experiments depending nonlinearly on intensity. Keeping the time delay fixed at 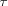 = 0 fs the overlap region of the two partial beams can be varied by changing the overlap angle. This causes different transverse fluence distributions. A nominally complete overlap of the two partial beams is depicted in Fig. 8 ▸(*b*). Now, an irregular modulation with maximum fluence values up to 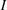 = 0.30 J mm^−2^ is obtained which is twice as high as for the separated case in Fig. 7 ▸(*b*), but significantly lower than in the just discussed case.

**Figure 9:**
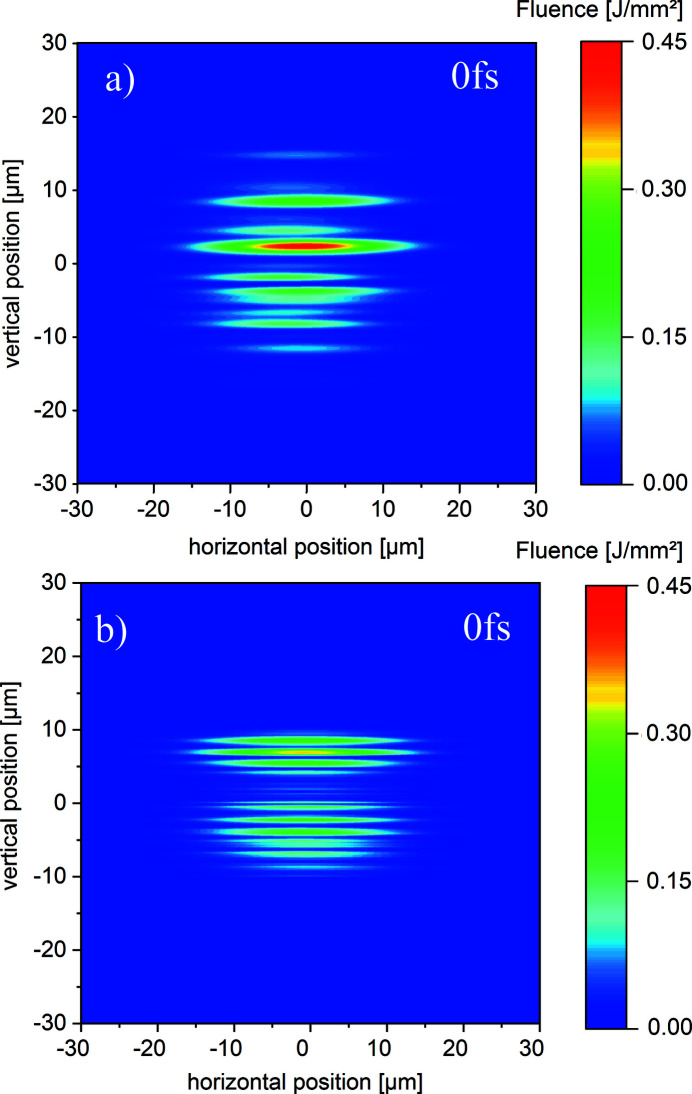
Including the two off-set mirrors OM1 and OM2 to the set-up yields these transverse profiles on an imaging detector located at the focal spot at *z* = 972 m split by the SDU overlapping the intense parts in (*a*). The focal spot for a complete overlap is depicted in (*b*) using one possible lens configuration in the HED tunnel.

Until now, only the CRLs and the SDU were included in the propagation path. According to Table 1 ▸ and Fig. 4 ▸, two additional off-set mirrors are located at 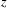 = 290 m and at 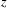 = 301 m behind the SASE2 undulator. Fig. 9 ▸ shows the case depicted in Fig. 8 ▸ including the impact of the off-set mirrors. By comparison of both figures the influence of the off-set mirrors seems to be small compared with the influence of the mirrors of the SDU. The fluence distribution at the target position is very similar to the one without considering OM1 and OM2. In Fig. 9 ▸(*a*) a maximum fluence of 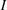 = 0.45 J mm^−2^ is obtained, again similar to the one of Fig. 8 ▸(*a*). The quality of the wavefront is thus not significantly affected by OM1 and OM2.

**Figure 10:**
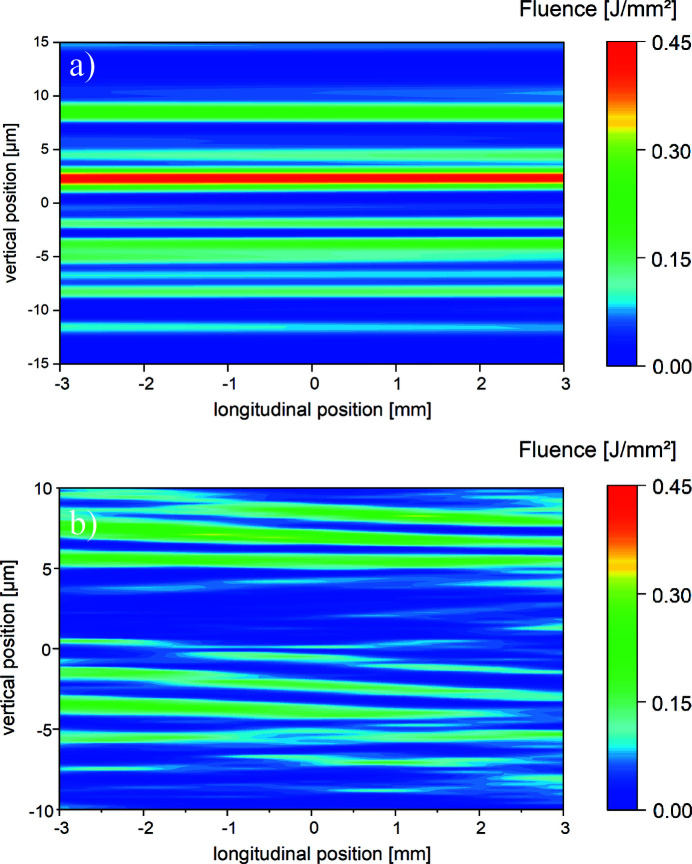
Vertical cuts for τ = 0 fs at *x* = 0 for 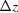 = ±3 mm around the focal spot at *z* = 972 m using real mirrors including two offset mirrors for one possible lens configuration in the HED tunnel. In Fig. 10 ▸(*a*) only the intense parts are overlapped and Fig. 10 ▸(*b*) displays a nominally full overlap.

The Rayleigh length for the simulated lens configuration is 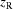 = 5 m. Thus, the user has plenty of room to place their target along the optical axis. Therefore, the impact of optics imperfection should also be studied in propagation direction around the focal spot. We calculate the vertical fluence distribution for 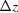 = ±3 mm around the geometrical spot position at 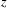 = 972 m for the same overlap region as depicted in Fig. 9 ▸. The vertical cut of Fig. 9 ▸(*a*) is displayed in Fig. 10 ▸(*a*). The distortions of the wavefront in longitudinal direction seem to be very constant for this partial overlap of the beams. For a full overlap the vertical cut is depicted in Fig. 10 ▸(*b*). Since the fringes outside this area have low fluence, only the overlap region is depicted in Fig. 10 ▸. This allows a closer inspection of the interesting part in the real case. For the simulated beam properties the full beam overlap produces rapidly fluctuating intensities while for a partial overlap the intensities in propagation direction remain constant. In a real experiment, it is thus important to overlap only the intense parts of the partial beams where the fluence modulation in the longitudinal direction is not changing strongly. SASE-induced shot-to-shot fluctuation will of course have an additional impact. The ideal case corresponding to Fig. 10 ▸(*b*) and the horizontal cuts at 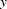 = 0 are added to Figs. A2.2 and A2.3 in the supporting information. In the ideal case the interference fringes are symmetrically and equally distributed in the overlap region which spreads over ±10 µm along the vertical axis *y*.

### 4.2. Influence of the real mirror profiles on a measurement of the coherence time τ_c_   

**Figure 11:**
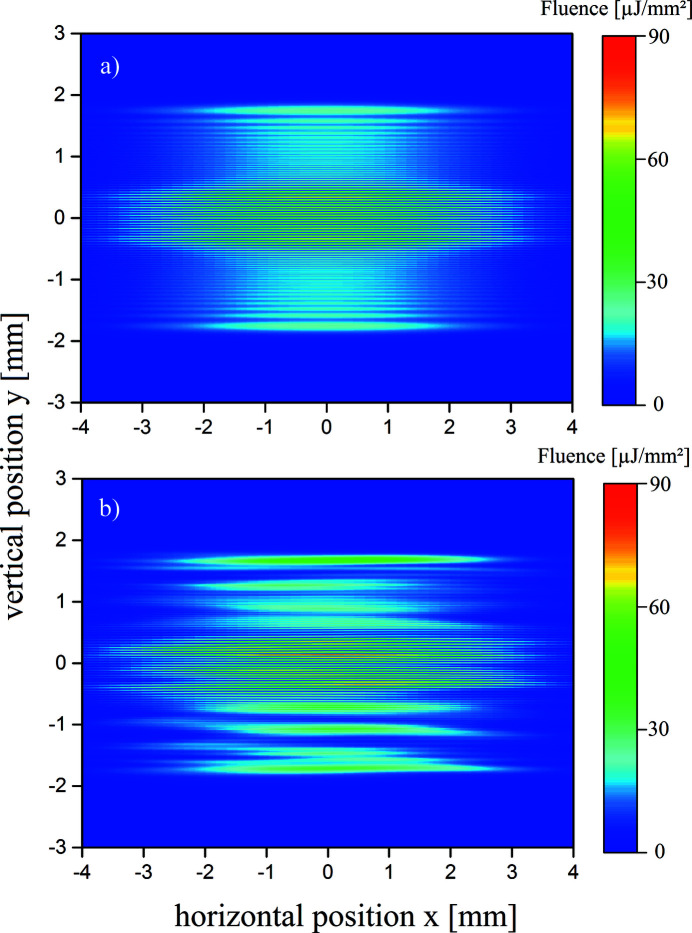
The transverse profile on an imaging detector at *z* = 972 m for (*a*) ideal and (*b*) real mirrors and an overlap of 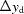 = 0.75 mm (20% of the beam profile).

For an investigation of the coherence properties of the XFEL pulses, which is important for the spatial profile during spatial and temporal beam overlap at the target, the partial beams depicted in Fig. 6 ▸ have to be overlapped. For a measurement of the longitudinal coherence, the overlap angle is fixed and the partial beams are delayed by different times τ. Fig. 11 ▸ shows the transverse profile for an overlap of 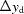 = 0.75 mm which corresponds to 20% of the beam profile for the ideal [Fig. 11 ▸(*a*)] and the real case [Fig. 11 ▸(*b*)]. This is achieved by an overlap angle of 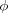 = 5.8 µrad according to equations (9) and (10). Here, the temporal delay is 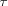 = 0. For a better visualization, vertical cuts at 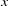 = 0 are shown in Fig. A3.1 in the supporting information for the ideal and in Fig. A3.2 for the real cases, respectively.

**Figure 12:**
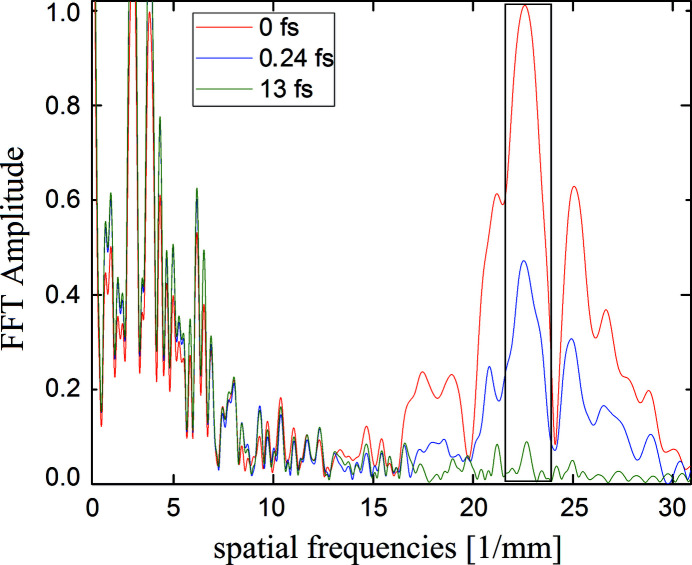
Normalized FFT for real mirrors. The black vertical line marks the area that is integrated in order to calculate the correlation function.

In the case of ideal mirror surfaces a clear modulation can be seen in the overlap region. Following equation (8), a visibility of 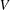 = 0.96 is calculated for 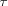 = 0 fs. This modulation decreases with increasing delays. For 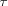 = 0.24 fs the visibility reduces to 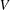 = 0.43, Fig. A3.1b, and for 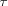 = 13 fs the visibility completely vanishes, Fig. A3.1c. For the real surfaces the vertical cut at 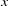 = 0 through the transverse interference distribution is characterized by asymmetric interference patterns as can be seen in Fig. A3.2. An estimation of the visibility by equation (8) yields 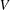 = 0.94 for 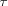 = 0 fs, 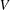 = 0.40 for 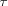 = 0.24 fs, and for 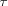 = 13 fs the visibility vanishes as in the ideal case. To obtain a meaningful evaluation, the spatial FFT of the interference patterns have to be calculated. In equation (11) the time correlation function 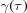 is directly connected to the spatial frequencies of the interference patterns caused only by the overlap itself. According to equation (10) the displayed interference patterns show a spatial period of 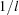 = 23 periods mm^−1^. The FFTs of the vertical cuts of the transverse fluence distribution are depicted in Fig. 12 ▸ for delays of 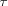 = 0 fs, 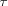 = 0.24 fs and 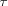 = 13 fs using real mirrors. The ideal case is depicted in Fig. A3.3 in the supporting information. From Fig. A3.3, it is obvious that the fringes which occur due to diffraction at the edges of BS and RC appear at spatial frequencies below 10 periods mm^−1^. The pattern caused by two-beam interference appear at 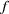 = 23 periods mm^−1^ as expected. This holds also in the case of real mirror surfaces in Fig. 12 ▸. The spatial frequencies resulting from the height deviations of the surfaces appear at spatial frequencies of less than 6 periods mm^−1^. That is in good agreement with equation (3) where the scattered spatial frequencies appear below 4 periods mm^−1^ due to the height profile of the beam splitter. According to equation (8), a visibility of 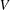 = 0.43 was calculated for ideal mirrors for a time delay of 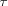 = 0.24 fs which is in good agreement with the blue curve in Fig. A3.3. The contribution of the spatial frequencies of the two-beam interference is normalized to the maximum value which is at 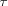 = 0 fs delay. With increasing delay, the height of the peak decreases. Since the contribution of the two-beam interference is not a δ function, as expected in equation (11), it is not sufficient to take only the maxima of the peaks into account, but one should integrate over the marked area around the peak as shown in Fig. 12 ▸.

**Figure 13:**
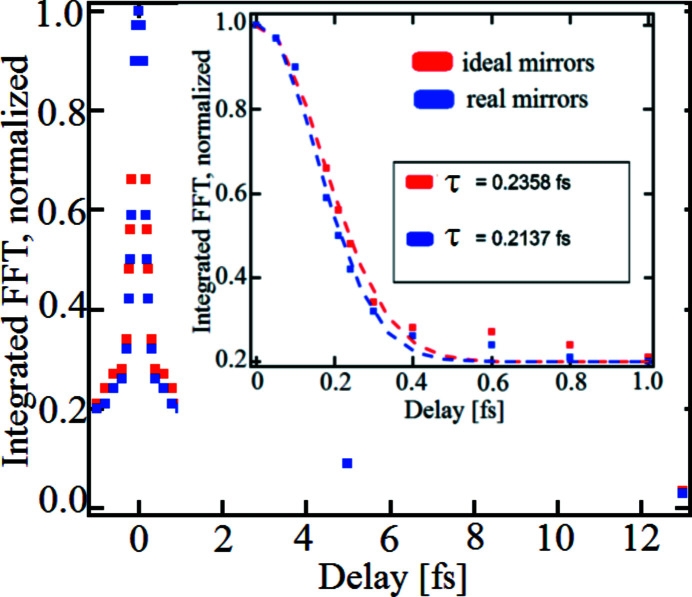
The simulated measurement of the temporal coherence properties of the SASE pulses for the ideal case (red labels) and the real case (blue labels). A Gaussian function with a constant background of 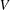 = 0.2 fit to values for delays between 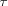 = 0 fs and 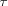 = 1 fs (inset) yields a coherence time of 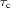 = 0.235 fs (HWHM) for the ideal case and 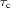 = 0.213 fs (HWHM) for the real case.

Fig. 13 ▸ displays the values of the integrals for delays between 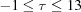
 fs. The red dots indicate a simulated measurement of the temporal coherence with ideal mirrors and the blue dots denote the case using real mirrors. The results show the typical characteristics of the temporal coherence properties of SASE pulses, a sharp spike around 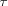 = 0 fs and a long tail. The inset of Fig. 13 ▸ shows a fit by a Gaussian function with a constant background of 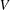 = 0.2 on a time scale of 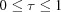 fs to the simulated correlation function. An evaluation of these graphs yields a coherence time of 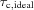 = 0.235 fs (HWHM) for ideal surfaces and 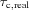 = 0.213 fs (HWHM) for real surfaces. Accordingly, the systematic error caused by the wavefront distortion amounts to only 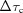 = 0.022 fs which corresponds to a relative deviation of 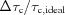 = 9.4%. In an earlier report, where only one-dimensional height profiles of only BS and RC were taken into account, we found a relative deviation between ideal and non-ideal surfaces of only 2.1% (Roling *et al.*, 2014*b* ▸). Thus, the two-dimensional height profiles of all eight mirrors increases the relative deviation, as expected. This deviation can, however, still be tolerated because it is smaller than pulse-to-pulse fluctuations of the coherence of the SASE pulses (see below).

**Figure 14:**
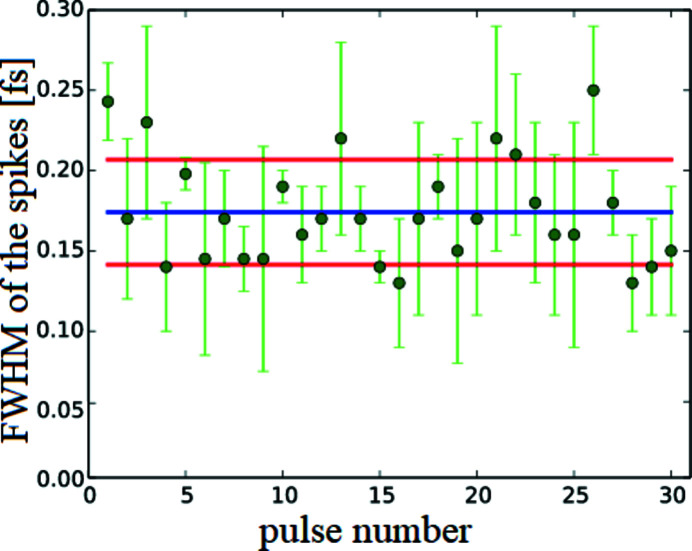
Averaged FWHM values of the spikes for different SASE pulses (green) are depicted. In addition, the ensemble averaged value for all SASE pulses (blue) is shown which is 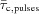 = 0.174 fs. The red lines mark the standard deviation of 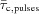 which is 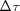 = 0.034 fs. This figure thus exemplifies the shot-to-shot fluctuation of the theoretically calculated SASE pulses (Saldin *et al.*, 1999 ▸).

The error of the measurement resulting from real mirrors is of the order of the deviation of the spikes widths in Fig. 5 ▸(*a*). As described earlier, the width of a spike (FWHM) is approximately the coherence time (FWHM). For this particular simulation the spikes widths yield a coherence time of 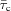 = (0.239 ± 0.024) fs. Hence, the error in the simulated measurement of the coherence time caused by the real mirror surfaces does not exceed the standard deviation of the spikes. In comparison, a measured uncertainty caused by the shot-to-shot fluctuations of the SASE pulses of 8% and 17% for wavelengths between 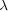 = 8 nm and 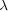 = 32 nm was observed (Roling *et al.*, 2011 ▸). To evaluate the pulse-to-pulse deviations theoretically, we investigate the shot-to-shot fluctuations of different SASE pulses for photon energies of 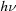 = 4.96 keV generated by the code *FAST* (Saldin *et al.*, 1999 ▸). The pulses are generated under the present operating conditions of the SASE2 undulator and start from shot noise. This induces different power distributions, and shot-to-shot fluctuations can be simulated. The fluctuations are investigated by taking the mean value of all spikes of a pulse weighted by the integral of the intensity of each spike. Fig. 14 ▸ shows the temporal widths of the spikes obtained for 30 independent pulse simulations. For each simulated pulse the average of the coherence times with their standard deviations are depicted as green dots and error bars. The spike duration 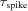 scatters in a range between 0.13 fs and 0.25 fs. The pulse number 1 is used for the propagation simulation and is already discussed. All pulses are generated under the present operating conditions of the European XFEL (Schneidmiller & Yurkov, 2017 ▸). The pulses show different deviations from their mean value. Some pulses fluctuate strongly while other pulses indicate peak widths with less fluctuations. To determine the shot-to-shot fluctuations of the theoretically calculated pulses, the average value of all pulses is depicted in Fig. 14 ▸ (blue line) along with their standard deviation (red lines). This yields an ensemble averaged coherence time of 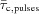 = (0.174 ± 0.034) fs. The statistical error of the coherence time of the SASE pulses is 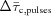 = 0.034 fs and 50% larger than the systematic error (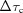 = 0.022 fs) caused by the real mirror surfaces. Hence, a measurement of the coherence properties of the SASE pulses by the SDU with real mirrors is meaningful. Further, the spatial overlap of both pulses in a pump/probe experiment will cause a 3D optical modulation of the intensity as long as both pulses are temporally overlapped.

## 5. Summary   

This paper describes a detailed simulation of the influence of all eight real mirrors of the novel split-and-delay unit on the unfocused and the focused beam profile for hard X-ray pump/X-ray probe experiments at the European XFEL. In addition, the influence of these mirrors on a measurement of the coherence is discussed. The time-dependent code *SRW* (Chubar *et al.*, 2008 ▸; Samoylova *et al.*, 2011 ▸) has proven its ability for this kind of simulation. The focusing elements in the HED beamline will be compound refractive lenses whose wavefront distortions are small compared with those of the grazing-incidence mirrors inside the SDU. The formation of interference fringes generated for time delays below the coherence time enhances the peak fluence values between *I* = 0.45 J mm^−2^ for a narrow overlap and *I* = 0.30 J mm^−2^ for a full overlap, which is an increase by 200 to 400% compared with the case without spatio-temporal coherence effects and ideal mirror surfaces. The two additional off-set mirrors OM1 and OM2 change the fluence distribution in and around the spot but the quality of the wavefront is not affected.

Wavefront distortions resulting from mirror imperfections lead to considerable perturbations of the interference patterns. Nevertheless, the relevant fringes for evaluating the coherence properties can be filtered by means of a Fourier transformation of the fluence distribution measured on a detector. In this way, coherence times of 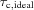 = 0.235 fs (HWHM) and 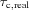 = 0.213 fs (HWHM) are found for ideal and real mirrors, respectively, resulting in a difference of 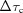 = 9.4%. In an earlier report, where only one-dimensional height profiles of the beam splitter and the recombiner were taken into account, we found a deviation of 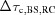 = 2.1% (Roling *et al.*, 2014*b* ▸). Hence, the three-dimensional topography of all eight mirrors increase the deviation but the systematic error of the measurement of 9.4% can be tolerated since the shot-to-shot fluctuations of the temporal coherence of the SASE pulses are larger. The simulations show that the topography of the mirrors of the SDU is good enough to support X-ray pump/X-ray probe experiments with high peak intensities.

## Figures and Tables

**Table 1:** The positions of all optical components for the HED beamline at the SASE2 undulator CRL2 and CRL3 will be used alternatively. The beam diameter is calculated using the set-up of Fig. 4 ▸ (Nakatsumi *et al.*, 2014 ▸).

Optical element	Position	Focal length	Beam diameter (FWHM)	Diameter (FWHM) without CRLs
CRL1	*z* = 229 m	*f* = 131–140 m	0.93 mm	–
Off-set mirror 1	*z* = 290 m	–	0.74 mm	1.18 mm
Off-set mirror 2	*z* = 301 m	–	0.71 mm	1.24 mm
Distribution mirror	*z* = 390 m	–	Neglected	
SDU	*z* = 846–852 m	–	0.99 mm	3.27 mm
CRL2	*z* = 857 m	*f* = 84–86 m	1.08 mm	–
CRL3	*z* = 962 m	*f* = 7–9 m	1.28 mm	–
Experiment/spot	*z* = 972 m	–	20 µm	3.89 mm
